# Data-Driven Reduction of External Load Variables in Indoor Team Sports Using Local Positioning System

**DOI:** 10.3390/jfmk11030249

**Published:** 2026-06-25

**Authors:** Christos Kokkotis, Ioannis Kansizoglou, Dimitrios Pantazis, Alexandra Avloniti, Dimitrios Balampanos, Panagiotis Foteinakis, Theodoros Stampoulis, Maria Protopapa, Alexandros Dendrinos, Panagiotis Aggelakis, Nikolaos Zaras, Paraskevi Malliou, Maria Michalopoulou, Antonios Gasteratos, Athanasios Chatzinikolaou

**Affiliations:** 1 Department of Occupational Therapy, School of Physical Education, Sport Science and Occupational Therapy, Democritus University of Thrace, 69100 Komotini, Greece; ckokkoti@ot.duth.gr (C.K.); iokansiz@ot.duth.gr (I.K.); tstampou@ot.duth.gr (T.S.); 2 Department of Physical Education and Sport Science, School of Physical Education, Sport Science and Occupational Therapy, Democritus University of Thrace, 69100 Komotini, Greece; dpantazi@phyed.duth.gr (D.P.); alavloni@phyed.duth.gr (A.A.); dimibala10@phyed.duth.gr (D.B.); pfotinak@phyed.duth.gr (P.F.); mprotopa@phyed.duth.gr (M.P.); adendrin@phyed.duth.gr (A.D.); pangelak@phyed.duth.gr (P.A.); nzaras@phyed.duth.gr (N.Z.); pmalliou@phyed.duth.gr (P.M.); michal@phyed.duth.gr (M.M.); 3 Department of Production and Management Engineering, School of Engineering, Democritus University of Thrace, 67132 Xanthi, Greece; agaster@pme.duth.gr

**Keywords:** external load, local positioning system, unsupervised learning, K-means clustering, external-load profiling, dimensionality reduction

## Abstract

**Objectives:** Local positioning systems (LPSs) used in indoor team sports generate a large number of external load variables, often exceeding practical monitoring capacity. The redundancy and overlap among these variables make it difficult to identify the most informative metrics for performance analysis and load management. This study aimed to reduce the dimensionality of external load variables derived from LPS data and to identify data-driven external-load observation profiles using principal component analysis and clustering techniques. **Methods:** A total of 188 observations from indoor team sports (basketball, handball, and futsal) were analyzed. Continuous external load variables were standardized and subjected to principal component analysis (PCA), with component retention based on a ≥90% cumulative explained variance threshold. K-means clustering was applied in both the full standardized feature space and the PCA-reduced space. The optimal number of clusters was determined using silhouette analysis and the elbow method. Agreement between clustering solutions was assessed using Adjusted Rand Index (ARI) and Normalized Mutual Information (NMI). Cluster characteristics were further examined using descriptive statistics and variable separation analysis. **Results:** The first two principal components explained 53.7% of the total variance, representing high-intensity external load and neuromuscular load dimensions, while 12 components were required to exceed 90% cumulative explained variance. Clustering analysis consistently identified three moderately separated clusters in both the full and PCA-reduced spaces. The PCA-based solution demonstrated improved separation (silhouette = 0.362) compared to the full-space solution (silhouette = 0.319). Agreement between clustering approaches was high (ARI = 0.981; NMI = 0.971), indicating that dimensionality reduction largely preserved the main clustering structure within the analyzed dataset. The most discriminative variables included jump load, acceleration load, metabolic power, and anaerobic activity distance. **Conclusions:** A large set of external load variables can be reduced into interpretable latent dimensions that support exploratory external-load profile identification. The combination of PCA and clustering provides an exploratory and structure-preserving framework for summarizing complex external-load datasets and identifying latent load dimensions. These findings may assist future monitoring strategies; however, the practical utility of the identified profiles requires prospective validation before implementation in training-load management.

## 1. Introduction

Historically, monitoring training load has been essential to designing training structures such as the annual training cycle, mesocycles, microcycles, training sessions, and drills [1,2,3,4,5,6,7,8,9]. Over the years, sports scientists have used tools such as rating of perceived exertion, heart rate indices, and work output indices [10,11,12,13,14,15,16]. The need to record and monitor training load led to the development of systems for tracking internal and external load, which, in combination with individualized capabilities, led to adaptations of the human biological system in response to training stimuli [17,18]. In recent years, high-technology systems have been developed for data recording and analysis. These systems are based on inertial measurement units (IMUs), local positioning systems (LPSs), and global positioning systems (GPSs), with the first two increasingly used as preferred methods in indoor team sports [16,19,20,21,22,23].

Load monitoring plays a dual role in sports science. On the one hand, it helps distribute work within training cycles to maximize athletic performance; on the other hand, by managing load, it aims to reduce the risk of injury [5,23,24,25,26]. Essentially, it seeks to answer how much training is sufficient to stimulate biological adaptations at a higher level of homeostasis while maintaining a low likelihood of injury. In recent years, with technological advancements, IMU and LPSs used in indoor team sports have enabled analysis of training load across the three movement axes, within intensity zones, and through quantified metrics such as total distance and training load. Compared with the recent past, when subjective measurements and internal load indices were evaluated, the number of variables that can serve as criteria for monitoring load has increased significantly.

The use of LPS and IMU systems in indoor sports after 2017, combined with advances in artificial intelligence and data analytics, has led to a substantial increase in the number of external load variables that can be monitored. Modern tracking systems can generate dozens of metrics simultaneously, capturing different aspects of movement, intensity, and mechanical load [27,28]. While this enhances the capacity to quantify performance, it also creates a practical challenge, as many of these variables are highly correlated and provide overlapping information [29]. As a result, practitioners often face an excess of data without clear guidance on which variables are most informative. Traditional approaches frequently rely on arbitrary variable selection and are limited in their ability to handle high-dimensional and interdependent data. In parallel with the expansion of tracking technologies, machine learning approaches have increasingly been applied in sports performance analysis [30].

Supervised learning methods have mainly been used for prediction tasks such as injury risk estimation, fatigue detection, match outcome prediction, and player classification. Ferraz et al. highlighted that recent research has increasingly focused on predicting performance, fatigue, and injuries using tracking-system data and combined external and internal load metrics [23]. Similarly, studies in team sports have employed supervised models to estimate perceived exertion, wellness status, and performance outcomes from GPS and LPS data [31]. Despite the growing application of predictive analytics, most supervised approaches focus on outcome prediction rather than on understanding the underlying structure and relationships among load variables [32,33]. In parallel, several studies have applied principal component analysis (PCA), clustering methods, and other unsupervised approaches to identify playing styles, tactical patterns, and latent performance structures in team sports. Recent reviews have shown that PCA is the most frequently used dimensionality-reduction technique in sports analytics and is often combined with clustering algorithms to classify teams according to playing style characteristics and tactical behaviors [34,35]. Previous applications have primarily focused on match statistics, event-based indicators, and tactical-performance variables, particularly in soccer, with substantially less attention given to external-load data derived from athlete-tracking technologies [34,35]. Furthermore, methodological heterogeneity in feature selection, preprocessing procedures, and reporting practices has limited the comparability and generalizability of findings across studies [34,35]. Consequently, less attention has been given to dimensionality reduction and the identification of latent performance constructs through unsupervised and exploratory approaches such as principal component analysis and clustering applied specifically to high-dimensional LPS-derived external-load datasets in indoor team sports. In this context, data-driven methods such as principal component analysis and clustering offer a systematic way to reduce dimensionality, identify underlying performance structures, and support exploratory external-load profile identification [34,35].

In recent years, in indoor sports, many studies have monitored variables such as accelerations, decelerations, training load based on these two, and distance covered in intensity zones. The two major categories of variables are those related to participation time and expressing the intensity of athletic activity, and those expressing an overall load, calculated as cumulative load [16,20,22,23]. Thus, the question arises as to whether it is useful to monitor all load indicators and how sports scientists should use them. In the literature to date, sports scientists, based on theoretical backgrounds, arbitrarily select monitoring variables. However, the relationships among the variables have not yet been investigated. Examining the relationships between variables and grouping them may reduce the number of parameters to monitor, making data monitoring and interpretation easier, supporting exploratory grouping of external-load observations, and, by extension, a better training structure.

The research hypothesis of the study was that the high-dimensional external-load variables provided by an indoor local positioning system contain redundant and interrelated information that can be summarized into a smaller number of latent dimensions. Accordingly, this study aimed to reduce the dimensionality of external training-load data obtained from a local positioning system in indoor team sports by combining correlation analysis, principal component analysis, and clustering methods. Specifically, the study sought to (i) identify core latent external-load dimensions and (ii) explore whether observation-level external-load profiles could be identified to support more interpretable monitoring and future hypothesis generation. Importantly, the purpose of the present study was not to develop athlete-classification models or training-prescription systems, but rather to explore the latent structure of LPS-derived external-load variables and generate interpretable hypotheses for future research.

## 2. Materials and Methods

### 2.1. Experimental Design

**Figure 1:**
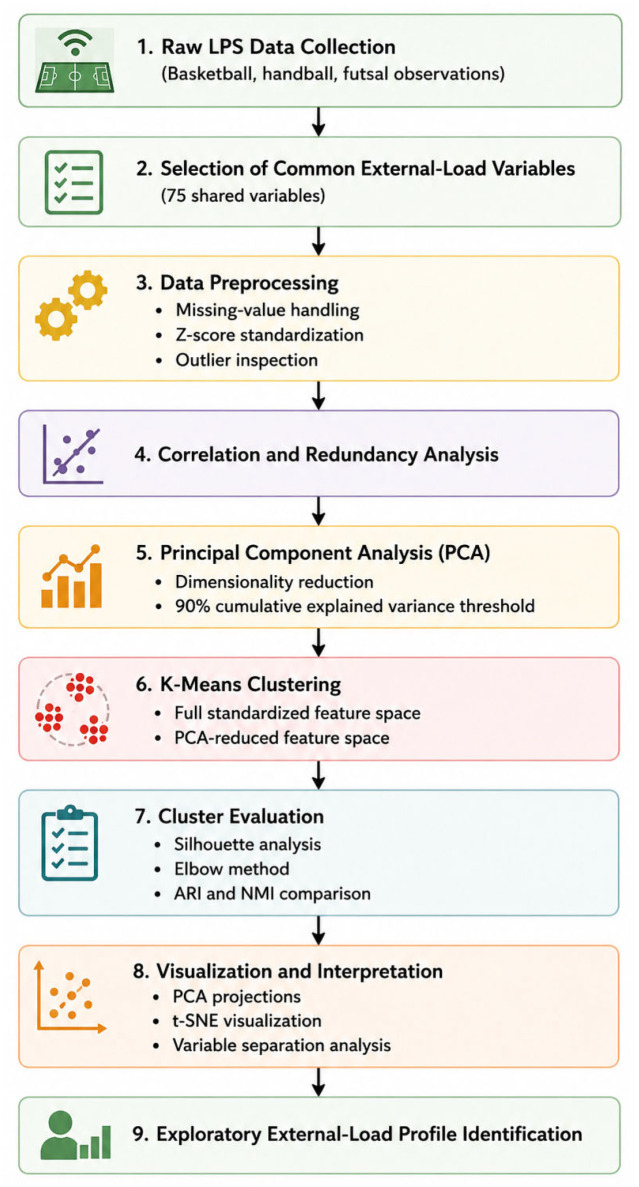
Schematic overview of the analytical workflow used in the present study. The pipeline included LPS data collection, preprocessing and standardization of external-load variables, dimensionality reduction using principal component analysis, clustering in both full and PCA-reduced spaces, cluster evaluation, and exploratory external-load profile interpretation.

The central methodological aim was to determine whether the large set of variables provided by the LPS could be reduced into a smaller number of interpretable external-load dimensions. Therefore, the analysis did not use a predefined target variable or supervised classification outcome. Instead, the study applied an unsupervised analytical framework designed to identify latent relationships among variables, reduce redundancy, and identify exploratory observation-level external-load profiles (Figure 1).

This approach was selected because practitioners are often required to interpret numerous overlapping external-load indicators, many of which may reflect similar physiological or mechanical demands. Therefore, the present study adopted an observational, data-driven design to investigate the underlying structure of external-load variables derived from a local positioning system (LPS) in indoor team sports. External-load data were collected under match-play conditions from basketball, futsal, and handball athletes. Specifically, two matches were conducted for each sport, resulting in a total of six analyzed matches. The participating teams were local competitive teams from the wider university region, and all participants were active athletes regularly engaged in organized training and competition. In total, 188 player observations were recorded. Data collection took place during the competitive period of each sport, specifically during weeks without additional match obligations, while athletes maintained their regular training schedules. This approach was adopted to ensure that the recorded external-load responses reflected representative match-play demands while minimizing the potential influence of fixture congestion or accumulated competitive fatigue.

Inclusion criteria were active participation in organized basketball, handball, or futsal; absence of injury at the time of data collection; and full participation in the monitored match-play conditions. Observations with incomplete, corrupted, or technically invalid tracking data were excluded. The study was approved by the Institutional Ethics Committee of the Department of Physical Education and Sport Science, Democritus University of Thrace (Protocol No: DUTH/EHDE/38826/966; approval date: 31 January 2025), and all participants provided written informed consent before participation.

All games were performed under official or official-like competitive conditions according to the standard rules of each sport. Although movement demands naturally differed between basketball, handball, and futsal, all observations represented intermittent indoor team-sport activity characterized by repeated accelerations, decelerations, changes in direction, and high-intensity actions.

### 2.2. Local Positioning System Data Collection

External load was monitored using a Local Positioning System (Kinexon GMBH, Munich, Germany) suitable for indoor team-sport environments. The system consists of 18 antennas mounted on the ceiling of the University Indoor Sports Facility, a central computer unit that functions as the main hub for communication between the antennas and the transmitters, and for data storage and processing. The system is based on Ultra-Wideband radio signal technology and achieves positional accuracy of <10 cm. The data frequency is 20 Hz. During the measurements, the researchers monitored the data in real time, which allowed them to prevent potential data loss due to technical issues. The athletes wore sensors which, for handball and futsal, were placed in a special vest between the shoulder blades, while for basketball players they were attached with a special clip at hip level, close to the athletes’ center of mass. The Kinexon sensor, in addition to communicating with the 18 antennas, contains an accelerometer (100 Hz), magnetometer, and gyroscope (200 Hz), enabling it to record movements along the three axes of motion. LPS and inertial-based technologies allow practitioners to quantify external load in sports with multidirectional, intermittent, and highly variable movement patterns. In the present analysis, the resulting dataset contained about 200 variables. However, because the study included games from three different sports, data were recorded and analyzed only for the 76 common variables.

### 2.3. Dataset

The final dataset comprised 188 observations from indoor team-sport athletes. Each observation represented an external load profile derived from LPS monitoring during match or match-like conditions. The dataset included athletes from basketball, handball, and futsal, enabling the analysis to capture a broad range of movement patterns in indoor sports. Because the objective of the study was exploratory dimensionality reduction rather than predictive modeling, observations from all sports were pooled into a common analytical framework. This approach was selected to maximize variability in external-load responses and to investigate whether common latent structures could emerge across indoor team-sport environments.

Only continuous external load variables were included in the principal component and clustering analyses. Variables containing only missing values were excluded, while missing values in the retained continuous variables were replaced using the median of the corresponding variable. This preprocessing step was used to preserve the sample size while minimizing the influence of extreme values. All continuous variables were then standardized before analysis to ensure that variables measured on different scales contributed equally to the multivariate procedures.

### 2.4. Statistical Analysis

All analyses were performed using Python 3.11. The statistical workflow was developed as a reproducible unsupervised machine-learning pipeline. First, the dataset was imported and cleaned. Empty fields were converted into missing values, and each variable was inspected to determine whether it should be treated as continuous or categorical. Continuous variables were retained for dimensionality reduction and clustering because the main objective was to investigate the structure of quantitative external load indicators.

Before multivariate analysis, all continuous variables were standardized using z-score normalization. Standardization was necessary because the dataset contained variables expressed in different units, such as meters, minutes, counts, watts, kilocalories, and arbitrary load units. Without scaling, variables with larger numerical ranges would have dominated the PCA and clustering solutions. Continuous variables were also inspected for extreme values after standardization. Observations were not removed solely because of statistical extremeness unless they reflected clear recording errors or corrupted tracking data, in order to preserve the natural variability of match-play demands.

Principal component analysis was then applied to the standardized dataset. PCA was used to identify latent dimensions underlying the original external load variables and to reduce redundancy among highly related metrics. All principal components were initially computed, and the cumulative explained variance was examined. The number of retained components was determined dynamically using a 90% cumulative explained variance threshold. This threshold was selected to ensure that the PCA-reduced representation retained most of the information contained in the original dataset while still achieving meaningful dimensionality reduction. As a result, the clustering analysis was performed on a reduced feature space that preserved the dominant variance structure of the original variables.

The correlation structure of the variables was inspected before PCA to confirm substantial inter-variable redundancy and multicollinearity, supporting the suitability of dimensionality-reduction analysis. The mean absolute inter-variable correlation was 0.351, while 166 variable pairs demonstrated strong correlations (|r| > 0.80). PCA suitability was additionally assessed using the Kaiser–Meyer–Olkin (KMO) measure and Bartlett’s test of sphericity. The overall KMO value was 0.831, indicating good sampling adequacy, while Bartlett’s test was significant (χ^2^ = 77,588.18, *p* < 0.001), confirming that the correlation matrix differed significantly from an identity matrix and supporting the suitability of the dataset for PCA.

The interpretation of each principal component was based on variable loadings. For each component, the variables with the highest absolute loadings were examined and used to assign a practical meaning to the new latent dimension. This step was essential because PCA generates mathematical components that require physiological and sport-specific interpretation. Components were therefore labeled according to the dominant type of external load represented by their strongest contributing variables.

K-means clustering was subsequently applied using two parallel approaches. First, clustering was performed in the full standardized feature space. Second, clustering was repeated in the PCA-reduced space using the retained principal components. This dual approach allowed comparison between the original high-dimensional variable structure and the reduced-dimensional representation. For both approaches, the number of clusters was evaluated across k values from 2 to 10. Cluster quality was assessed using inertia and the silhouette score. Inertia was used to examine within-cluster compactness through the elbow method, whereas the silhouette score was used to quantify the balance between within-cluster cohesion and between-cluster separation.

The optimal number of clusters was selected as the k value that maximized the silhouette score. After final clustering, the agreement between the full-space and PCA-space solutions was quantified using the Adjusted Rand Index and Normalized Mutual Information. These indices were used to determine whether dimensionality reduction preserved the underlying clustering structure. A high agreement between the two solutions would indicate that PCA reduced the number of variables without materially altering the main pattern of external-load profiles. K-means clustering was selected because the aim was to identify compact centroid-based groupings in a continuous standardized feature space and to allow direct comparison between clustering in the original and PCA-reduced spaces. However, because K-means assumes approximately spherical clusters and may be sensitive to initialization and outliers, the resulting profiles were interpreted as exploratory rather than definitive classifications. To improve reproducibility, clustering was performed using fixed random-state initialization, and sensitivity checks across multiple random seeds were used to examine cluster stability.

To support interpretation, cluster sizes, descriptive statistics, and variable separation scores were calculated. Variable separation was assessed using the mean absolute deviation of each cluster mean from the global mean. This analysis identified the variables that contributed most strongly to differences between clusters. In addition, t-SNE was used as a nonlinear dimensionality reduction method to project observations onto two dimensions and visually explore the spatial organization of the observations and clustering structure.

#### 2.4.1. PCA Interpretation Strategy

The PCA results were interpreted by examining the signed and absolute loadings of the original variables on each retained component. The first principal component was interpreted as a high-intensity external load dimension because it was mainly defined by anaerobic activity distance, high-speed distance and time, very-high acceleration load, sprints, accelerations, and exertions. The second component reflected a contrast between medium acceleration load and low-speed volume on one side, and deceleration and jump-load variables on the other. This component was therefore interpreted as a neuromuscular load versus a low-intensity volume dimension.

The third principal component primarily represented overall exposure and workload volume, as it was characterized by time on the playing field, total distance, low-speed distance and time, metabolic work, and low-intensity acceleration-load variables. The fourth component represented maximal speed and sprint-related performance, with high loadings for very-high sprints, high accelerations, very-high and extreme speed distance, and high-speed activity. The fifth component reflected a metabolic-mechanical contrast, with opposing contributions from maximal metabolic power, jump-related variables, and high acceleration-load variables.

This interpretation enabled the PCA solution to be translated into interpretable external-load dimensions rather than being treated solely as a mathematical reduction procedure. In this way, the study identified not only fewer variables but also broader external load constructs that may be easier for practitioners to monitor and interpret.

#### 2.4.2. Cluster Interpretation Strategy

Clusters were interpreted by combining three sources of information: their positions in the PCA space, the variables with the highest separation scores, and their consistency across the full-space and PCA-reduced clustering solutions. This integrated approach was used to avoid interpreting clusters based on a single statistical output. Instead, each cluster was interpreted as an exploratory external-load profile emerging from multiple complementary analyses.

The PCA plots were used to determine whether clusters were separated mainly along PC1, PC2, or both. The variable separation analysis was then used to identify which original external load metrics differed most between groups. Finally, the agreement between the full-space and PCA-space solutions was examined to determine whether the identified profiles were stable after dimensionality reduction. Together, these analyses provided a complete explanation of how the reduced PCA dimensions, original external load variables, and final external-load profiles were connected.

## 3. Results

### 3.1. PCA and Dimensional Structure of the Data

**Figure 2:**
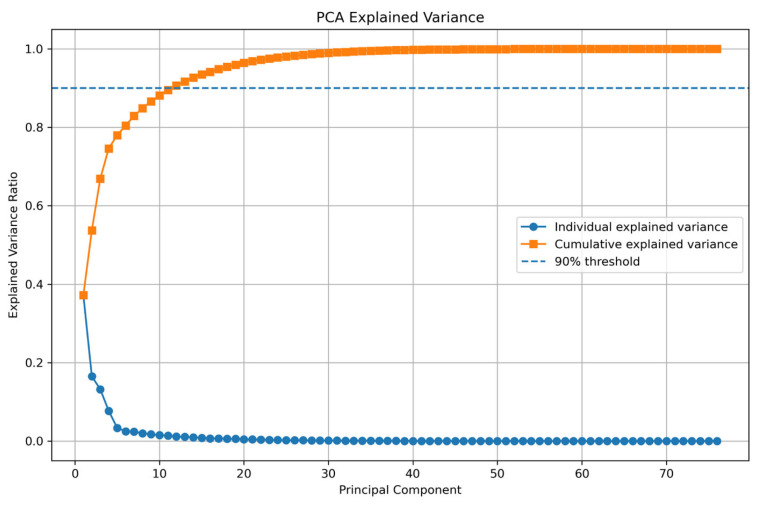
PCA explained variance and cumulative explained variance.

Principal component analysis revealed that the first principal component (PC1) explained 37.2% of the total variance, while the second component (PC2) accounted for 16.5%. Together, the first two components explained 53.7% of the total variance, whereas the first three components explained 66.9%. To reach the predefined threshold of 90% cumulative explained variance, 12 principal components were required, accounting for 90.6% of the variance (Table 1). These findings indicate that, despite the large number of original variables, a substantial proportion of the variance structure in the dataset is captured by a relatively small number of latent dimensions (Figure 2).

To further interpret the latent structure of the data, the loadings of the original variables on each principal component were examined (Appendix A). Because the first five principal components accounted for approximately 78.0% of the total variance and provided the clearest practical interpretation, these components were summarized in Table 2.

Although 12 principal components were retained to satisfy the predefined ≥90% cumulative explained variance threshold, the interpretation focused primarily on the first five components. These first five components accounted for approximately 78.0% of the total variance and represented the most interpretable and practically meaningful dimensions of the dataset. In contrast, components 6–12 each contributed only a small additional proportion of variance, ranging approximately from 1.1% to 2.5%, and were retained mainly to preserve the overall structure of the data for PCA-based clustering. Therefore, the first five components were used for substantive interpretation, whereas all 12 retained components were used in the reduced-space clustering analysis to ensure that the dimensionality-reduced representation preserved at least 90% of the original information.

### 3.2. Cluster Visualization in PCA Space

**Figure 3:**
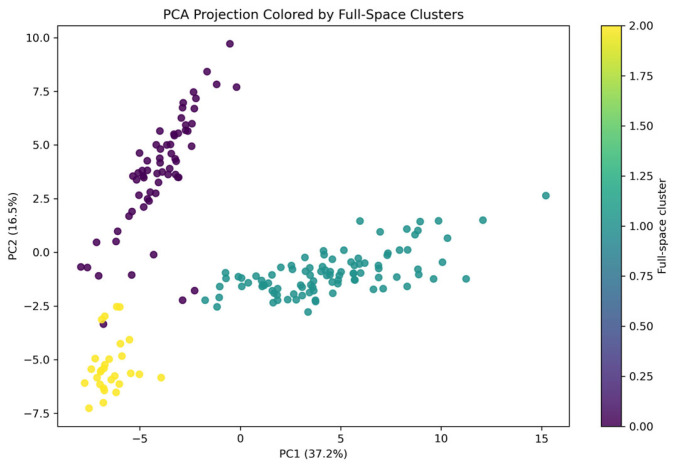
Projection of observations onto the first two principal components (PC1 and PC2), colored according to clustering performed in the full standardized feature space. PC1 primarily reflects high-intensity external load characteristics, whereas PC2 is mainly associated with neuromuscular and low-intensity volume dimensions. The visualization illustrates the moderate separation of the three exploratory external-load profiles.

**Figure 4:**
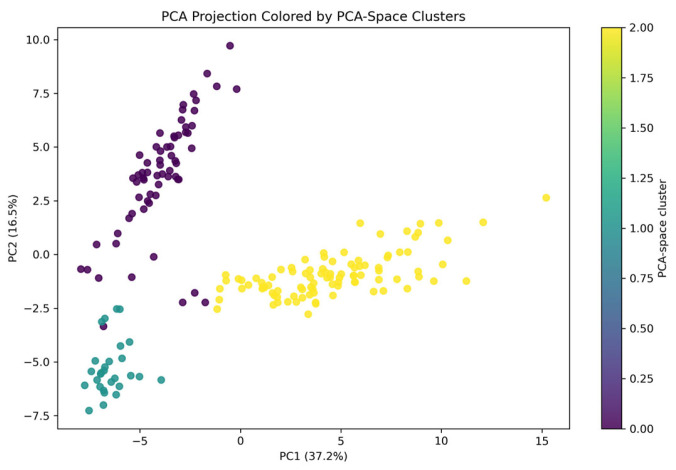
Projection of observations onto the first two principal components (PC1 and PC2), colored according to clustering performed in the PCA-reduced feature space. The PCA-space clustering solution demonstrated a similar grouping structure to the full-space clustering solution, supporting the preservation of the main clustering structure after dimensionality reduction.

Projection of the observations onto the first two principal components revealed a visually apparent but moderately separated three-cluster pattern (Figure 3 and Figure 4). One cluster was primarily located along positive values of PC1, whereas the remaining two clusters were positioned at more negative values of PC1 and were mainly differentiated along PC2. This geometric configuration suggests that PC1 represents a dominant axis of variation, likely associated with overall load or intensity, while PC2 contributes to the differentiation of sub-profiles within lower-intensity observations.

### 3.3. Clustering Results and Model Comparison

The PCA adequacy diagnostics supported the suitability of the dataset for dimensionality reduction, with a KMO value of 0.831 and a significant Bartlett’s test of sphericity (*p* < 0.001).

The analysis of 188 observations indicated that the optimal clustering solution comprised three clusters in both the full standardized feature space and the PCA-reduced space.

PCA-reduced solution showed moderately improved separation, with a higher silhouette score (0.362) than the full-space solution (0.319). These values indicate moderate rather than strong cluster separation, which is expected in biological and sports-performance datasets where movement demands often vary along continuous rather than sharply discrete patterns.

Agreement between the two clustering solutions was high, with an Adjusted Rand Index (ARI) of 0.981 and a Normalized Mutual Information (NMI) of 0.971. These findings suggest that dimensionality reduction preserved the main clustering structure within the present dataset. (Table 3 and Table 4).

### 3.4. Selection of the Number of Clusters

**Figure 5:**
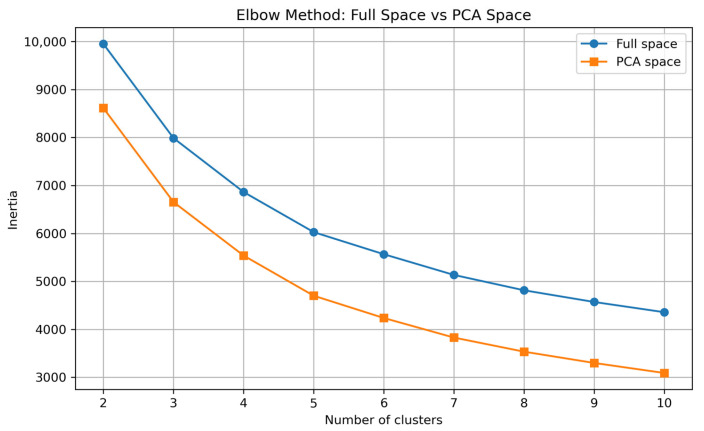
Elbow-method comparison between clustering performed in the full standardized feature space and the PCA-reduced space. Inertia decreased progressively as the number of clusters increased, with a noticeable inflection point around k = 3–4 clusters, supporting the selection of a three-cluster solution.

The elbow method showed a gradual decrease in inertia as the number of clusters increased from k = 2 to k = 10 (Figure 5). The largest reduction occurred in the initial steps, with a noticeable inflection point around k = 3–4 clusters. This suggests that adding more clusters beyond this point yields diminishing returns in within-cluster compactness. In the PCA-reduced space, inertia values were consistently lower than in the full feature space, as expected due to the projection onto a lower-dimensional subspace that retains most of the informative variance while reducing noise.

**Figure 6:**
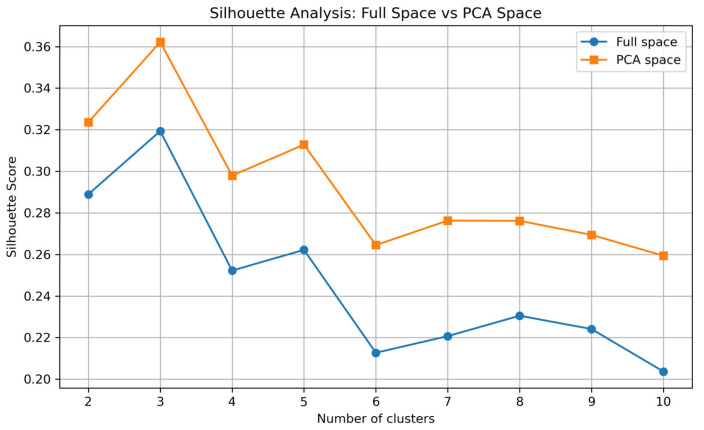
Silhouette-score comparison between clustering performed in the full standardized feature space and the PCA-reduced space across different numbers of clusters (k = 2–10). The highest silhouette values were observed at k = 3 in both analytical spaces, indicating moderate but optimal cluster separation for the three-cluster solution.

Silhouette analysis provided a clearer criterion for selecting the optimal number of clusters. The highest silhouette score in the full space (0.319) and in the PCA-reduced space (0.362) was observed at k = 3 (Figure 6). Moreover, the PCA-based solution consistently outperformed the full-space solution across most k values, indicating that dimensionality reduction improved the expression of the cluster structure. Based on these findings, a three-cluster solution was selected for further interpretation.

The cluster-size distribution was highly consistent between the full-space and PCA-space solutions. In the full-space clustering, the three clusters included 64, 96, and 28 observations, whereas in the PCA-space clustering, the corresponding clusters included 65, 95, and 28 observations (Table 5 and Table 6). Therefore, the only difference between the two solutions was the reassignment of a single observation between the two larger clusters, while the smallest cluster remained unchanged. This further supports the stability of the clustering structure and indicates that dimensionality reduction did not meaningfully alter the underlying grouping of observations.

Furthermore, it should be noted that cluster labels (e.g., Cluster 0, 1, and 2) are arbitrarily assigned by the clustering algorithm and do not correspond directly between the full-space and PCA-space solutions. The numerical labeling depends on the initial centroid assignment and the order in which clusters are identified during the K-means procedure. Therefore, cluster indices should not be interpreted as representing the same group across different clustering approaches. Instead, comparisons between solutions should be based on cluster characteristics and membership rather than label numbering.

### 3.5. t-SNE Visualization of Clustering Structure

**Figure 7:**
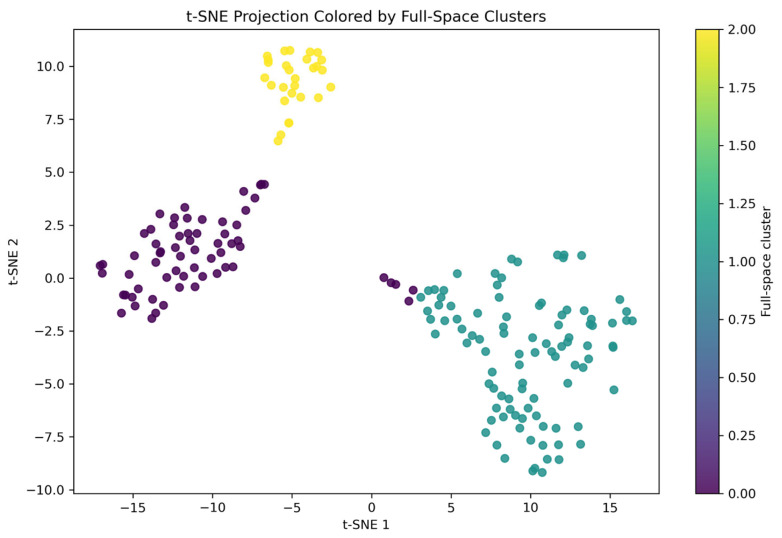
Two-dimensional t-SNE projection of observations colored according to clustering performed in the full standardized feature space. The figure provides a qualitative visualization of the clustering structure and should not be interpreted as an independent validation of cluster validity.

**Figure 8:**
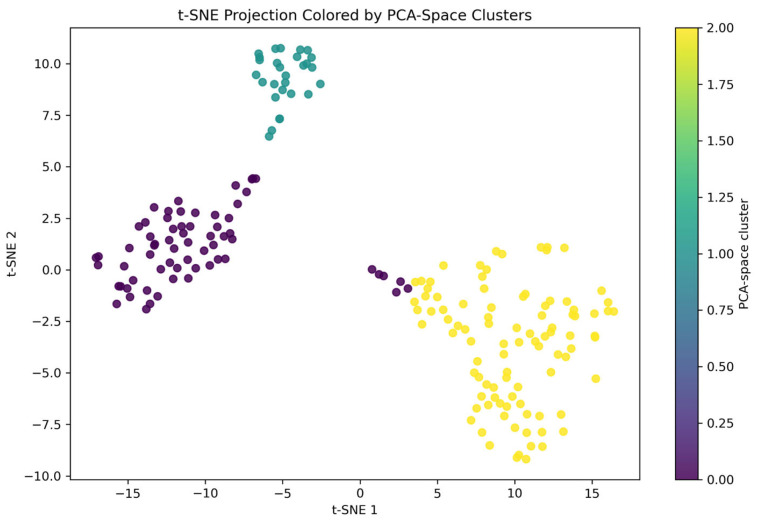
Two-dimensional t-SNE projection of observations colored according to clustering performed in the PCA-reduced feature space. The visualization demonstrates a similar spatial grouping pattern to the full-space clustering solution while serving only as a qualitative representation of the data structure.

The t-SNE projections were used as nonlinear two-dimensional visualizations of the data structure. In both representations, the observations showed a visually apparent three-group pattern with limited overlap. However, t-SNE was used only as a qualitative visualization tool and not as an independent validation method for cluster validity. Because nonlinear projection methods may exaggerate apparent separation depending on hyperparameter selection, these plots should be interpreted cautiously and only as supportive visual summaries of the clustering results (Figure 7 and Figure 8).

### 3.6. Variables Separating the Clusters

The analysis of separating variables was based on the mean absolute deviation of each cluster mean from the global mean of the corresponding variable (Table 7 and Table 8). In both clustering approaches, Jump Load (J) emerged as the most influential variable, with a separation score exceeding 5293 units. Other highly discriminative variables included high-intensity metrics such as Acceleration Load in the very high zone, maximal metabolic power, Acceleration Load in the low zone, and Anaerobic Activity distance.

The fact that the same variables appeared among the top contributors in both the full-space and PCA-based clustering solutions indicates that cluster interpretation is stable and not dependent on the chosen analytical space.

### 3.7. Integrated Interpretation

The principal component structure and clustering results were closely aligned, indicating that the identified clusters emerged directly from the dataset’s dominant latent dimensions. Specifically, the primary separation between clusters occurred along PC1, which represents high-intensity external load, while secondary differentiation was observed along PC2, reflecting neuromuscular load characteristics.

This interpretation is further supported by the variable separation analysis, in which the most discriminative variables, such as jump load, acceleration load, and anaerobic activity, correspond to those with the highest loadings on the first two principal components. These findings suggest that the clustering structure is driven by a combination of intensity-related and neuromuscular performance factors. Although the three profiles demonstrated consistent statistical separation within the present dataset, they should not be interpreted as distinct athlete types. Instead, they represent exploratory groupings within a continuous external-load spectrum.

## 4. Discussion

In an era of rapid technological advances in load monitoring that generate large volumes of data, the primary aim of this study was to reduce the dimensionality of external load variables derived from an LPS and to explore observation-level external-load profiles using an unsupervised data-driven approach [16,23,27,28,36]. The findings suggest that highly interrelated external-load variables can be summarized into a smaller number of interpretable latent dimensions, which may support more efficient and transparent load monitoring in indoor team sports [27,28,29].

An important methodological implication of the present study is that dimensionality reduction, when applied carefully, preserves the data’s intrinsic structure rather than distorting it [27,29]. This was demonstrated by directly comparing clustering solutions obtained in the full standardized feature space and the PCA-reduced space. The exceptionally high agreement between the two solutions (ARI = 0.981; NMI = 0.971), combined with the nearly identical cluster-size distributions and minimal reassignment of observations, indicates that the reduced representation retains the essential characteristics of the original dataset. This finding is relevant for both data analysts and practitioners, as it suggests that dimensionality reduction can simplify complex datasets while preserving the main structure of the analyzed data [28,29,37,38]. In this context, PCA should be considered an exploratory structure-reduction technique rather than a validation method for athlete profiling [29,38]. Consequently, the comparison between full-space and reduced-space clustering supports the internal consistency of the identified observation-level profiles within the present dataset, but external validation remains necessary.

The PCA results provided a clear and interpretable representation of the latent structure of external load [29,38]. Specifically, the first principal component, explaining 37.2% of the variance, was dominated by high-intensity variables such as anaerobic activity, high-speed running, sprint actions, and very high acceleration load. This component can be interpreted as a global high-intensity external load axis, reflecting the overall explosive and metabolically demanding aspects of performance. The second principal component, explaining an additional 16.5% of the variance, captured a distinct dimension related to neuromuscular load and movement dynamics, characterized by variables such as decelerations and jump-related metrics, in contrast to lower-intensity volume indicators. Together, these components accounted for more than half of the total variance, indicating that external load in indoor team sports can be largely described by a combination of intensity-driven and neuromuscular factors [16,22,29,38]. It is important to clarify that the distinction between component retention and component interpretation is central to the methodological approach of this study. Twelve principal components were retained to exceed the 90% explained variance threshold and to ensure that the PCA-reduced clustering solution preserved the essential structure of the original dataset. However, the first five components explained approximately 78% of the total variance and captured the most coherent and practically interpretable external-load dimensions. The remaining components each contributed only a small additional percentage of variance and appeared to represent more specific or residual aspects of movement behavior. For this reason, they were included in the clustering procedure to avoid unnecessary information loss, but the practical interpretation was concentrated on the first five components. An additional consideration is that the identified latent dimensions emerged from the statistical structure of the analyzed dataset and may therefore vary across sports, competitive levels, monitoring technologies, and analytical procedures. Consequently, the dimensions identified in the present study should not be assumed to represent universal constructs of external load, but rather preliminary data-driven representations that require replication in independent samples.

Importantly, the clustering structure was directly aligned with these principal components. The primary separation between clusters occurred along PC1, suggesting that differences in high-intensity load represent the dominant factor distinguishing performance profiles. Secondary differentiation along PC2 further separated observations based on neuromuscular demands and movement characteristics. This strong alignment between PCA and clustering suggests that the identified clusters reflect structured patterns within the dataset rather than purely random algorithmic partitioning. For data analysts, this highlights the importance of combining unsupervised learning techniques to move beyond purely descriptive metrics and toward interpretable latent structures [28,35,38]. The three-cluster solution may be interpreted as three exploratory external-load profiles rather than as arbitrary statistical groups. The smallest cluster (approximately 15% of observations) appears to represent the most demanding high-intensity profile, characterized by markedly elevated acceleration-load, anaerobic activity, and sprint-related variables. In contrast, the largest cluster was associated with more moderate and cumulative workload characteristics, including total distance and low-speed activity. The remaining cluster demonstrated a mixed neuromuscular profile with stronger contributions from jump-related and deceleration variables. This distribution suggests that the identified profiles may reflect distinct movement-demand patterns rather than simple differences in overall activity volume. Specifically, based on the PCA structure, the variable separation analysis, and the spatial distribution of the observations, the first profile appears to represent observations characterized by higher high-intensity external load. This profile is mainly associated with greater values in variables related to anaerobic activity, high-speed distance, sprinting actions, and very high acceleration load, corresponding primarily to the positive direction of PC1. From a practical perspective, this profile may reflect athletes or match periods with greater explosive and metabolically demanding activity. A second profile appears to represent observations with comparatively lower high-intensity load but greater contribution from overall volume and lower-intensity activity. This profile is consistent with the contribution of PC3 and variables such as total distance, low-speed distance, time on the playing field, and metabolic work. Although these observations may not be characterized by the highest explosive demands, they still represent meaningful cumulative workload and should not be interpreted as having low importance from a monitoring perspective. The third profile appears to be distinguished more strongly by neuromuscular and mechanical load characteristics, including jump load, decelerations, and acceleration-related variables. This profile is particularly relevant in indoor team sports, where repeated braking, changes in direction, jumping, and short accelerative efforts are central to performance demands. Therefore, the three profiles may be understood as representing different combinations of high-intensity output, cumulative workload, and neuromuscular-mechanical stress. Importantly, these profiles were observed in both the full standardized feature space and the PCA-reduced space, with nearly identical cluster-size distributions and very high agreement indices. This supports the view that the profiles are not artifacts of dimensionality reduction, but consistent patterns within the analyzed dataset.

The variable separation analysis further reinforced this interpretation. Jump Load emerged as the most discriminative variable across both clustering approaches, followed by acceleration load, metabolic power, and anaerobic activity distance. These variables are all associated with high mechanical and metabolic stress, indicating that cluster differentiation is primarily driven by high-intensity and neuromuscular load characteristics [16,20,22,29]. The consistency of these findings across both analytical spaces confirms the robustness of the results and suggests that a small subset of variables can effectively capture the dataset’s structure [28,29,37]. The separation of the two axes and their contribution to explaining the data is consistent with the nature of indoor team sports. These sports are, by definition, intermittent, with periods of high intensity followed by periods of lower intensity [11,14,16]. A key characteristic of these sports is the change in direction, accelerations and decelerations, and, overall, high-intensity actions across all three movement axes [16,21,39,40]. Between them, the sports present differences; for example, in basketball, more actions are required in the vertical axis, expressed through parameters such as jump load, whereas in futsal and handball, greater emphasis is placed on the horizontal axis, where accelerations and decelerations over short time intervals are the decisive performance factors [16,20,22,29,39,40].

The t-SNE projections provided an additional qualitative visualization of the clustering structure in a nonlinear low-dimensional space [41]. In both representations, the observations showed a visually apparent three-group pattern with relatively limited overlap. However, because t-SNE is primarily a visualization technique and may exaggerate apparent separation depending on hyperparameter selection, these projections should not be interpreted as independent validation of cluster validity [28,37].

From a research and applied perspective, many studies in recent years have focused on identifying the key parameters that determine athletic performance and, ultimately, match outcomes [16,23,35]. The parameters that are particularly recognized in futsal and handball are the ability to perform a high number of accelerations and decelerations within short time periods, as well as the ability to perform sprints [39,40]. However, it is also clear that indoor load monitoring systems show high validity for positional data and for variables related to the total number of accelerations, decelerations, and high-intensity actions, while they demonstrate lower accuracy in determining maximal accelerations, decelerations, and maximal speed [42,43,44,45]. The results of the present study suggest exploratory external-load profiles that also incorporate parameters related to athletes’ maximal capacities. The inclusion of athletes from different sports helps increase the sample’s dispersion and supports the identification of exploratory external-load profiles. Essentially, to our knowledge, few studies have explored observation-level external-load profiling in indoor team sports using LPS-derived data [42,43,44,45]. Nevertheless, although the results are in line with the nature of these sports, they should be interpreted with caution, as the axes derived include parameters with relatively low validity [42,43,44,45].

From a practical perspective, the present findings should be interpreted as hypothesis-generating rather than immediately actionable. Although the identified dimensions and profiles may assist practitioners in summarizing complex monitoring datasets, their usefulness for decision-making, athlete management, and training prescription has not yet been prospectively demonstrated. Modern tracking systems generate numerous external load variables, many of which provide redundant information [28,29,37]. Monitoring all available metrics is not only impractical but may also obscure meaningful insights. The present study suggests that these variables can be summarized into a smaller number of interpretable dimensions, primarily high-intensity load, neuromuscular load, and overall workload volume, thereby allowing practitioners to focus on the most informative aspects of performance [28,29,37]. Building on this and on the basis that load monitoring has dual significance in sport, as it is involved in both maximizing performance and reducing injury risk, and in guiding the return-to-play process for injured athletes [3,5,6,46], the development of hybrid parameters, calculated from multiple variables without losing the original information, might better explain the relationship between load and outcomes [27,28,38]. However, the practical value of the identified dimensions remains to be established. Longitudinal monitoring of these three dimensions within long-term training planning, together with the examination of their relationships with performance outcomes, injury occurrence, and markers of maladaptation or overtraining, may help determine whether the proposed framework provides meaningful advantages for athlete monitoring and decision-making.

The parameters used so far in the literature relate to accelerations, decelerations, and high-intensity actions and stem mainly from studies in soccer and American football, and to a lesser extent in futsal and handball, while research in basketball is almost nonexistent [16,19,20,22,24,25,26,39,40]. Based on the present study’s data, it appears that the identified latent dimensions group high-intensity actions within common external-load structures, creating specific profiles; however, variables of moderate or low intensity, which also contribute to the athletes’ overall load, likewise help explain the data. Longitudinal monitoring of load within each athlete profile may potentially highlight different parameters associated with both performance maximization and injury risk reduction [6,18,46]. The results of this preliminary study can contribute to the development of exploratory external-load profiles in indoor team sports. Nevertheless, for these profiles to be used prospectively, field studies are needed to demonstrate their practical utility. At this stage, it is recommended that they be monitored alongside the indicators already used by coaches, and that they complement current monitoring practice only if longitudinal studies confirm their importance.

Additionally, the identification of different external-load patterns suggests that observations may respond differently to similar movement demands based on their load characteristics. However, the present findings should not be interpreted as validated individualized monitoring or training-prescription models. Instead, they provide an exploratory framework that may support future hypothesis generation and longitudinal investigation of individualized responses to training and competition demands. From a data science perspective, this reinforces the value of unsupervised learning methods for discovering hidden patterns in complex performance data [28,29,35,38].

Despite these strengths, several limitations should be acknowledged. First, the dataset included observations from multiple indoor sports, which may introduce variability related to sport-specific movement demands [16,39,40]. While this broadens the ecological scope of the findings, future studies could investigate whether similar dimensional structures emerge within individual sports. It should be mentioned that the inclusion of athletes from different indoor sports was intentional, as the objective of the study was not to derive sport-specific profiles, but rather to investigate whether common latent external-load structures emerge across indoor team sports characterized by intermittent multidirectional activity. Therefore, the identified dimensions should be interpreted as exploratory shared indoor-sport load patterns within the present dataset, rather than as generalized or sport-specific performance signatures. Second, the analysis was limited to external load variables and did not incorporate internal load measures such as heart rate, perceived exertion, or physiological responses. Integrating internal and external load data could provide a more comprehensive understanding of athlete stress and adaptation [3,6,12,13]. Third, in the model used to explain the data and to create external-load profiles, variables were included whose validity is debatable. Parameters such as maximal acceleration, deceleration, and speed show lower validity when assessed with LPSs [42,43,44,45]. Nevertheless, they were used because they represent commonly monitored variables in indoor team-sport load analysis, and the technology used to evaluate them is currently among the most commonly used and practically applicable technologies available for indoor monitoring.

Furthermore, the unsupervised nature of the analysis means that the identified clusters were not directly linked to performance outcomes or injury risk. Although the clusters may reflect interpretable external-load structures, their practical significance would be strengthened by examining their relationship with match performance, fatigue, or injury incidence [3,5,6,46]. Future research should therefore explore the predictive value of these profiles and assess their applicability in real-world performance and health monitoring contexts. Finally, while PCA and clustering effectively reduced dimensionality and improved interpretability, alternative machine learning approaches, such as nonlinear dimensionality reduction or model-based clustering, may provide additional insights into the structure of external load data [27,35,41]. Future work could compare different analytical techniques and evaluate their relative performance in identifying meaningful external-load profiles.

Additionally, the sample size of the present study was relatively modest compared with the dimensionality of the dataset, which may influence PCA stability, clustering reproducibility, and external generalizability. Nevertheless, the PCA adequacy metrics and the stability analyses across random seeds supported the internal consistency of the dimensionality-reduction and clustering procedures within the present dataset. Furthermore, no external validation cohort was available, and repeated-measures longitudinal modeling was not performed. Consequently, the temporal stability and individual responsiveness of the identified external-load profiles remain unknown and should be investigated in future longitudinal studies.

Moreover, K-means clustering assumes compact and approximately spherical cluster structures and may be sensitive to initialization procedures, outliers, and covariance structure. In addition, contextual variables such as playing position, tactical role, match status, opponent characteristics, and situational game demands were not incorporated into the present analysis. Future research should therefore examine whether the identified latent dimensions and exploratory external-load profiles remain stable when these contextual and sport-specific factors are considered. Furthermore, observations were analyzed at a single time point without repeated longitudinal measurements. Therefore, the stability of the identified dimensions and profiles across different phases of the season, training cycles, or competitive contexts remains unknown and should be examined in future longitudinal studies.

## 5. Conclusions

The present study suggests that high-dimensional external-load datasets obtained from indoor-sport local positioning system monitoring can be reduced into a smaller number of interpretable latent dimensions while preserving the main structure of the analyzed data. The combination of principal component analysis and clustering identified exploratory observation-level external-load profiles mainly characterized by high-intensity, neuromuscular, and cumulative workload dimensions. These findings support the potential usefulness of data-driven dimensionality-reduction approaches for simplifying complex monitoring datasets and improving the interpretability of external-load information in indoor team sports. However, the identified dimensions and profiles should be considered exploratory statistical constructs that describe patterns within the present dataset. Their translational value for athlete monitoring, performance optimization, injury prevention, and return-to-play decision-making remains to be established through prospective longitudinal validation studies. Future longitudinal and prospective studies are required to determine whether these latent dimensions and external-load profiles are associated with fatigue, recovery dynamics, injury risk, adaptation, or competitive performance outcomes.

## Figures and Tables

**Table 1:** Explained variance for the first 12 principal components.

PC	Explained Variance Ratio	Cumulative Explained Variance
PC1	0.372	0.372
PC2	0.165	0.537
PC3	0.132	0.669
PC4	0.077	0.746
PC5	0.034	0.780
PC6	0.025	0.805
PC7	0.024	0.829
PC8	0.020	0.849
PC9	0.017	0.866
PC10	0.015	0.881
PC11	0.014	0.895
PC12	0.011	0.906

**Table 2:** Top loadings and interpretation of principal components.

Principal Component	Top Contributing Variables	Interpretation
PC1	Anaerobic Activity (distance), Distance (high speed), Time (high speed), Acceleration Load (very high), Sprints, Exertions	High-intensity external load/explosive performance
PC2	Acceleration Load (medium), Distance (very low speed), Exertions (low), High Metabolic Power Distance, Decelerations (−), Jump Load (−)	Neuromuscular load vs. low-intensity volume balance
PC3	Time on field, Total distance, Low-speed activity, Metabolic work	Total workload volume/exposure
PC4	Sprints (very high), Accelerations (high), High-speed distance, Extreme speed	Maximal speed and sprint performance
PC5	Metabolic power (max) (−), Acceleration load (high), Jumps (−), Exertions	Metabolic vs. mechanical load trade-off

**Table 3:** Summary of clustering and PCA metrics.

Metric	Value
N observations	188
Best k-full space	3
Silhouette-full space	0.319
Best k-PCA space	3
Silhouette-PCA space	0.362
Adjusted Rand Index	0.981
Normalized Mutual Information	0.971
PCs retained for ≥90% variance	12

**Table 4:** K selection metrics across full and PCA spaces.

k	Inertia Full Space	Silhouette Full Space	Inertia PCA Space	Silhouette PCA Space
2	9949.168	0.289	8612.055	0.324
3	7987.396	0.319	6653.794	0.362
4	6861.741	0.252	5537.781	0.298
5	6027.549	0.262	4701.008	0.313
6	5553.446	0.213	4240.172	0.264
7	5134.894	0.221	3831.658	0.276
8	4810.997	0.231	3543.77	0.276
9	4564.802	0.224	3304.29	0.269
10	4349.493	0.204	3089.726	0.259

**Table 5:** Cluster sizes in full-space clustering.

Cluster Based on Full-Space	n
0	64
1	96
2	28

**Table 6:** Cluster sizes in PCA-space clustering.

Cluster Based on PCA-Space	n
0	65
1	28
2	95

**Table 7:** Top 10 separating variables for full-space clustering.

Variable	Separation Score
Jump Load (J)	5308.724
Acceleration Load (distance|very high) (m)	804.726
Metabolic Power (max.) (W)	725.389
Acceleration Load (distance|low) (m)	718.144
Anaerobic Activity (distance) (m)	625.423
Distance (speed|low) (m)	367.639
Distance (speed|very low) (m)	338.026
Distance (m)	320.137
Acceleration Load (distance|medium) (m)	319.937
Distance (speed|high) (m)	306.452

**Table 8:** Top 10 separating variables for PCA-space clustering.

Variable	Separation Score
Jump Load (J)	5292.885
Acceleration Load (distance|very high) (m)	805.356
Metabolic Power (max.) (W)	726.836
Acceleration Load (distance|low) (m)	709.185
Anaerobic Activity (distance) (m)	627.856
Distance (speed|low) (m)	360.088
Distance (speed|very low) (m)	334.147
Acceleration Load (distance|medium) (m)	314.287
Distance (speed|high) (m)	308.201
Distance (m)	303.045

## Data Availability

The data presented in this study are available on request from the corresponding author due to privacy and ethical reasons.

## Appendix A

**Table A1:** Loading of PCA factors.

Variable	Absolute Loading	PC	Signed Loading
Anaerobic Activity (distance) (m)	0.180186244	PC1	0.180186244
Time (speed|high) (min)	0.17797113	PC1	0.17797113
Distance (speed|high) (m)	0.177436499	PC1	0.177436499
Acceleration Load (distance|very high) (m)	0.177258075	PC1	0.177258075
Anaerobic Activity (time) (min)	0.176700023	PC1	0.176700023
Acceleration Load (time|very high) (min)	0.173824939	PC1	0.173824939
Accelerations (medium)	0.172228372	PC1	0.172228372
Acceleration Load (load|very high)	0.172151191	PC1	0.172151191
Sprints	0.171366581	PC1	0.171366581
Exertions	0.169843782	PC1	0.169843782
Decelerations (medium)	0.169797326	PC1	0.169797326
Accelerations	0.169559368	PC1	0.169559368
Sprints (low)	0.164161417	PC1	0.164161417
Exertions (high)	0.159947669	PC1	0.159947669
Accelerations (low)	0.158993591	PC1	0.158993591
Acceleration Load (distance|medium) (m)	0.228500493	PC2	0.228500493
Acceleration Load (load|medium)	0.227270879	PC2	0.227270879
Distance (speed|very low) (m)	0.218956026	PC2	0.218956026
Exertions (low)	0.216308743	PC2	0.216308743
Decelerations (low)	0.210980976	PC2	−0.210980976
Decelerations	0.209688108	PC2	−0.209688108
High Metabolic Power Distance (m)	0.190221178	PC2	0.190221178
Metabolic Power (time|very high) (min)	0.189455827	PC2	0.189455827
Jump Load per mass (J/kg)	0.183514863	PC2	−0.183514863
Jump Load (J)	0.18329944	PC2	−0.18329944
Metabolic Power (time|high) (min)	0.179887293	PC2	0.179887293
Metabolic Power per mass (Ø) (W/kg)	0.176990829	PC2	0.176990829
High Speed and Acceleration Distance (m)	0.173137274	PC2	0.173137274
Metabolic Power (time|medium) (min)	0.173126323	PC2	0.173126323
Jumps	0.168236406	PC2	−0.168236406
Time on Playing Field (min)	0.276823222	PC3	0.276823222
Distance (speed|low) (m)	0.257586637	PC3	0.257586637
Time (speed|low) (min)	0.249229231	PC3	0.249229231
Distance (m)	0.23588568	PC3	0.23588568
Metabolic Power (time|low) (min)	0.228809614	PC3	0.228809614
Metabolic Power (time|medium) (min)	0.213765753	PC3	0.213765753
Acceleration Load (time|low) (min)	0.207712241	PC3	0.207712241
Jumps	0.194261996	PC3	0.194261996
Metabolic Work (kcal)	0.191826131	PC3	0.191826131
Acceleration Load (load|low)	0.187767757	PC3	0.187767757
Jump Load per mass (J/kg)	0.181096772	PC3	0.181096772
Decelerations (low)	0.181080405	PC3	0.181080405
Acceleration Load (distance|low) (m)	0.177815236	PC3	0.177815236
Jumps (medium)	0.176877155	PC3	0.176877155
Jump Load (J)	0.176232222	PC3	0.176232222
Sprints (very high)	0.251000957	PC4	0.251000957
Accelerations (high)	0.241007153	PC4	0.241007153
Distance (speed|very high) (m)	0.222930387	PC4	0.222930387
Time (speed|very high) (min)	0.22208839	PC4	0.22208839
Sprints (high)	0.219931469	PC4	0.219931469
Distance (speed|extreme) (m)	0.21876476	PC4	0.21876476
Time (speed|extreme) (min)	0.218065276	PC4	0.218065276
Decelerations (high)	0.208091214	PC4	0.208091214
High Speed and Acceleration Distance (m)	0.175336824	PC4	0.175336824
Changes in Direction (right)	0.170728357	PC4	0.170728357
High Speed and Acceleration Time (min)	0.169989373	PC4	0.169989373
Metabolic Work (kcal)	0.161277271	PC4	−0.161277271
Changes in Direction	0.160293327	PC4	0.160293327
Acceleration Load (time|medium) (min)	0.154930902	PC4	−0.154930902
Metabolic Power per mass (max.) (W/kg)	0.153463408	PC4	0.153463408
Metabolic Power (max.) (W)	0.332375743	PC5	−0.332375743
Acceleration Load (distance|high) (m)	0.332362863	PC5	0.332362863
Acceleration Load (load|high)	0.311590592	PC5	0.311590592
Jumps (extreme)	0.248749664	PC5	−0.248749664
Jumps (very high)	0.240368131	PC5	−0.240368131
Acceleration Load (time|high) (min)	0.23600281	PC5	0.23600281
Exertions (medium)	0.212565909	PC5	0.212565909
Metabolic Power per mass (max.) (W/kg)	0.199023285	PC5	−0.199023285
Acceleration Load (max.)	0.191590958	PC5	−0.191590958
Exertions (low)	0.171129178	PC5	0.171129178
Metabolic Work (kcal)	0.157334948	PC5	−0.157334948
Exertions (very high)	0.156139279	PC5	−0.156139279
Jumps (high)	0.155756842	PC5	−0.155756842
Acceleration Load (time|medium) (min)	0.14935359	PC5	−0.14935359
Acceleration (max.) (m/s^2^)	0.147534575	PC5	−0.147534575
Jumps (extreme)	0.299178981	PC6	0.299178981
Acceleration Load (distance|high) (m)	0.262458055	PC6	0.262458055
Changes in Direction	0.250490716	PC6	−0.250490716
Acceleration Load (load|high)	0.249580464	PC6	0.249580464
Changes in Direction (right)	0.247804594	PC6	−0.247804594
Jumps (very high)	0.235258336	PC6	0.235258336
Changes in Direction (left)	0.218477544	PC6	−0.218477544
Jumps (medium)	0.21556577	PC6	0.21556577
Jumps (high)	0.19786022	PC6	0.19786022
Sprints (very high)	0.194385957	PC6	0.194385957
Time (speed|extreme) (min)	0.186648273	PC6	0.186648273
Distance (speed|extreme) (m)	0.176460159	PC6	0.176460159
Decelerations (medium)	0.162380236	PC6	−0.162380236
Acceleration Load (time|high) (min)	0.159086482	PC6	0.159086482
Acceleration Load (max.)	0.152700453	PC6	0.152700453
Time (speed|extreme) (min)	0.416109288	PC7	0.416109288
Distance (speed|extreme) (m)	0.41167961	PC7	0.41167961
Sprints (very high)	0.297273566	PC7	0.297273566
Jumps (low)	0.255484822	PC7	−0.255484822
Jumps	0.230601757	PC7	−0.230601757
Jump Load (J)	0.228571217	PC7	−0.228571217
Decelerations (very high)	0.224581464	PC7	−0.224581464
Jump Load per mass (J/kg)	0.219848378	PC7	−0.219848378
High Metabolic Power Distance (m)	0.208062528	PC7	−0.208062528
Accelerations (very high)	0.197999577	PC7	−0.197999577
High Speed and Acceleration Distance (m)	0.183683957	PC7	−0.183683957
High Speed and Acceleration Time (min)	0.163929838	PC7	−0.163929838
Metabolic Power (time|very high) (min)	0.159906586	PC7	−0.159906586
Distance (speed|very high) (m)	0.109767154	PC7	−0.109767154
Time (speed|very high) (min)	0.094416715	PC7	−0.094416715
Jumps (low)	0.265244327	PC8	−0.265244327
Decelerations (very high)	0.262248742	PC8	−0.262248742
Distance (speed|extreme) (m)	0.256097537	PC8	−0.256097537
Time (speed|extreme) (min)	0.253068585	PC8	−0.253068585
Accelerations (high)	0.225418115	PC8	0.225418115
Sprints (high)	0.202124237	PC8	0.202124237
Jumps	0.201771573	PC8	−0.201771573
Sprints (medium)	0.195672542	PC8	0.195672542
Jump Load per mass (J/kg)	0.194411968	PC8	−0.194411968
Jump Load (J)	0.185280069	PC8	−0.185280069
Sprints (very high)	0.175290693	PC8	−0.175290693
Time (speed|very high) (min)	0.173369595	PC8	0.173369595
Metabolic Power per mass (Ø) (W/kg)	0.171816018	PC8	−0.171816018
Acceleration (max.) (m/s^2^)	0.16846313	PC8	0.16846313
Distance (speed|very high) (m)	0.161917011	PC8	0.161917011
Accelerations (very high)	0.301471959	PC9	−0.301471959
Changes in Direction (right)	0.269322869	PC9	0.269322869
Jumps (extreme)	0.25506698	PC9	0.25506698
Time (speed|very low) (min)	0.253600245	PC9	−0.253600245
Jumps (high)	0.250515286	PC9	0.250515286
Changes in Direction	0.242219718	PC9	0.242219718
Exertions (medium)	0.210247262	PC9	−0.210247262
Changes in Direction (left)	0.178573122	PC9	0.178573122
Metabolic Power per mass (Ø) (W/kg)	0.174724551	PC9	0.174724551
Jumps (low)	0.173007106	PC9	−0.173007106
Accelerations (high)	0.158398584	PC9	0.158398584
Distance (speed|extreme) (m)	0.155857094	PC9	−0.155857094
Time (speed|extreme) (min)	0.146856849	PC9	−0.146856849
Accelerations (low)	0.144489274	PC9	−0.144489274
Accelerations	0.141642254	PC9	−0.141642254
Accelerations (very high)	0.415723948	PC10	0.415723948
Jumps (high)	0.342097839	PC10	0.342097839
Changes in Direction (right)	0.2550451	PC10	0.2550451
Acceleration (max.) (m/s^2^)	0.236734305	PC10	0.236734305
Changes in Direction	0.234849544	PC10	0.234849544
Jumps (very high)	0.226969203	PC10	0.226969203
High Metabolic Power Distance (m)	0.206787903	PC10	−0.206787903
Changes in Direction (left)	0.179834826	PC10	0.179834826
Metabolic Power per mass (max.) (W/kg)	0.174130286	PC10	0.174130286
Deceleration (max.) (m/s^2^)	0.172706969	PC10	−0.172706969
Decelerations (very high)	0.165558306	PC10	0.165558306
Jumps (low)	0.159499287	PC10	−0.159499287
Metabolic Power (time|high) (min)	0.147615981	PC10	−0.147615981
Acceleration Load (distance|high) (m)	0.140839488	PC10	0.140839488
Exertions (low)	0.139669192	PC10	0.139669192
Jumps (very high)	0.383797674	PC11	−0.383797674
Jumps (extreme)	0.368966112	PC11	0.368966112
Acceleration Load (max.)	0.35481772	PC11	0.35481772
Jumps (high)	0.325967404	PC11	−0.325967404
Decelerations (very high)	0.207537494	PC11	0.207537494
Exertions (very high)	0.206261185	PC11	0.206261185
Deceleration (max.) (m/s^2^)	0.193390458	PC11	−0.193390458
Accelerations (very high)	0.177363691	PC11	0.177363691
Exertions (medium)	0.176328773	PC11	−0.176328773
Acceleration Load (Ø)	0.154091774	PC11	0.154091774
Accumulated Acceleration Load	0.149079486	PC11	0.149079486
Jumps (medium)	0.14038894	PC11	−0.14038894
Decelerations (high)	0.123144008	PC11	0.123144008
Jumps (low)	0.118695809	PC11	0.118695809
Exertions (high)	0.118217085	PC11	−0.118217085
Decelerations (very high)	0.4722633	PC12	0.4722633
Changes in Direction (right)	0.293978974	PC12	−0.293978974
Time (speed|very low) (min)	0.281194044	PC12	−0.281194044
Deceleration (max.) (m/s^2^)	0.256127691	PC12	−0.256127691
Distance (speed|very low) (m)	0.2540957	PC12	−0.2540957
Changes in Direction	0.252654717	PC12	−0.252654717
Jumps (extreme)	0.244032416	PC12	−0.244032416
Time (speed|low) (min)	0.182209765	PC12	0.182209765
Decelerations (high)	0.173949257	PC12	0.173949257
Decelerations (low)	0.172010259	PC12	0.172010259
Changes in Direction (left)	0.171898631	PC12	−0.171898631
Accelerations (very high)	0.159475465	PC12	−0.159475465
Decelerations	0.149115016	PC12	0.149115016
Acceleration (max.) (m/s^2^)	0.148845932	PC12	0.148845932
Accelerations (high)	0.130720575	PC12	0.130720575
